# 

**Published:** 1882-07

**Authors:** 


### CONTENTS.

### COMMUNICATIONS.

Functional Habitude........................................................... 315
Exposed Pulps—Treatment and Filling.................................. 323

### PROCEEDINGS.

Proceedings of the Michigan State Dental Society.......................... 330
Proceedings of the Eighteenth Annual Meeting of the Illinois State Dental Society.......................... 339
To the Profession .......................................................... 345
National Dental Association of the United States of America...... 351
American Dental Association.............................................. 351

### CORRESPONDENCE.

Dental Diplomas in the State of New York.............................. 353
Letter from Dr. E. G. Betty.................................................. 355

### SELECTIONS.

Action of Chloroform.......................................................... 355
American Medical Association.............................................. 356
Causes of Complaint Against Vaccination.............................. 357

### EDITORIAL.

The American Dental Association........................................ 359
Wisconsin Dental College Again.......................................... 360
Petition to the Municipal Council of Paris, France..... .............. 361
The Tail of the Beast.......................................................... 362
Going Abroad.................................................................... 364
American Dental Association.............................................. 364
Pennsylvania State Dental Society....................................... 364

